# Phage-microbe interactions may contribute to the population structure and dynamics of hydrothermal vent symbionts

**DOI:** 10.1093/ismeco/ycag022

**Published:** 2026-02-03

**Authors:** Michelle A Hauer, Katherine M Klier, Marguerite V Langwig, Karthik Anantharaman, Roxanne A Beinart

**Affiliations:** Graduate School of Oceanography, University of Rhode Island, Narragansett, RI 02882, United States; School of Marine and Environmental Affairs, University of Washington, Seattle, WA 98105, United States; Northwest Fisheries Science Center, National Oceanic and Atmospheric Administration, Seattle, WA 98112, United States; Department of Bacteriology, University of Wisconsin-Madison, Madison, WI 53706, United States; Freshwater and Marine Sciences Program, University of Wisconsin-Madison, Madison WI 53706, United States; Department of Bacteriology, University of Wisconsin-Madison, Madison, WI 53706, United States; Freshwater and Marine Sciences Program, University of Wisconsin-Madison, Madison WI 53706, United States; Department of Bacteriology, University of Wisconsin-Madison, Madison, WI 53706, United States; Department of Integrative Biology, University of Wisconsin-Madison, Madison WI 53706, United States; Department of Data Science and AI, Wadhwani School of Data Science and AI, Indian Institute of Technology Madras, Chennai, Tamil Nadu, 600036, India; Graduate School of Oceanography, University of Rhode Island, Narragansett, RI 02882, United States

**Keywords:** microbial symbiosis, hydrothermal vents, bacteriophage, Lau Basin, *Alviniconcha*, *Ifremeria*, *Bathymodiolus*

## Abstract

Deep-sea hydrothermal vent ecosystems are sustained by chemoautotrophic bacteria that symbiotically provide organic matter to their animal hosts through the oxidation of chemical reductants in vent fluids. Hydrothermal vents also support unique viral communities that often exhibit high host-specificity and frequently integrate into host genomes as prophages; however, little is known about the role of viruses in influencing the chemosynthetic symbionts of vent foundation fauna. Here, we present a comprehensive examination of contemporary lysogenic and lytic bacteriophage infections, auxiliary metabolic genes (AMGs), and CRISPR spacers associated with the intracellular bacterial endosymbionts of snails and mussels at hydrothermal vents in the Lau Basin (Tonga). Our investigation of contemporary phage infection among bacterial symbiont species and across distant vent locations indicated that each symbiont species interacts with different phage species across a large geographic range. Surprisingly, prophages were absent from almost all symbiont genomes, suggesting that phage interactions with intracellular symbionts may differ from free-living microbes at vents. Altogether, these findings suggest that chemosynthetic symbionts primarily interact with species-specific phages via lytic infections, which may ultimately be important to the composition and dynamics of symbiont populations.

## Introduction

Hydrothermal vents are remarkable habitats found at the ocean floor, where geothermally heated fluids are released through fissures in the Earth’s crust. These ecosystems are sustained by chemosynthetic bacteria and archaea, which use inorganic molecules expelled from the venting fluid, like hydrogen sulfide and methane, as an energy source to fix CO_2_ into organic matter. Chemosynthetic microbes at hydrothermal vents exist both as free-living in the water column, rocks, and sediments, as well as symbiotically with foundation species of animals like tubeworms, mussels, and snails, where they provide their hosts with vital nutrients via chemosynthesis [1]. Despite the crucial role of symbiotic bacteria in these ecosystems supporting key animal species, phages—viruses that infect bacteria—remain relatively poorly studied among animal-associated populations of chemosynthetic bacteria at hydrothermal vents. Addressing this issue is essential, as phages could play a key role in shaping symbiont populations and in regulating bacterial-animal symbioses.

Viruses are the most abundant biological entities in the oceans, playing a crucial role in bacterial diversity [2–10], biogeochemical cycling of our oceans [11], microbial food web dynamics [12], and in aiding microbial survival [13, 14] . Much of what is known about the role of viruses in influencing biogeochemistry and structuring bacterial communities comes specifically from lytic (i.e. virion-producing) phages, which infect and lyse their bacterial hosts. Lysis releases dissolved and particulate material, contributing to biogeochemical cycles and controlling bacterial population dynamics and community composition, e.g. by preventing any single bacterial strain from dominating an ecosystem [15]. Further, microevolution driven by interaction with lytic phages also contributes to strain-level bacterial population structure and variation. In the evolutionary arms race between bacteria and phages, bacteria have evolved CRISPR (clustered regularly interspaced short palindromic repeats) spacers, viral DNA fragments incorporated into bacterial genomes that serve as an immune memory and enable bacteria to recognize and prevent future invaders [16]. These spacers, therefore, represent a historic catalog of some of the past attempted infections of bacterial hosts. Studies have found that phages may influence bacterial population structure [17–20] including at the strain-level [21, 22], and there is ongoing research in the use of CRISPR as a hypervariable region for micro-evolution [23]. In contrast, lysogenic phages directly alter their host bacterium’s genome by inserting their own genetic material into the bacterium’s chromosome, becoming a “prophage” i.e. replicated through binary fission. Since a prophage requires use of its host bacterium’s machinery for its own replication, it can be advantageous for prophages to enhance their host fitness via auxiliary metabolic genes (AMGs) [13, 14]. These viral genes, originally derived from bacteria, can be used to modulate the host bacterium’s metabolic activities and contribute to biogeochemical processes by enhancing nutritional absorption, survival in unfavorable conditions, and pathogenicity [12, 24–31]. Altogether, phage–bacterium interactions, encompassing both ecological (i.e. ongoing infections) and evolutionary (i.e. past infections evident from CRISPR spacers) processes, can drive genomic and phenotypic variation within and among bacterial species.

Temperate phages can adopt a **lysogenic** infection mode, in which their genomes integrate into the bacterial chromosome and persist as **prophages**. Under certain conditions, they can transition into the **lytic** cycle, during which they excise (if integrated), replicate, and lyse their hosts. These temperate phages are notably abundant at hydrothermal vents relative to other marine habitats [32, 33], often harboring AMGs that modulate their host bacterium’s metabolism (e.g. nitrogen and sulfur metabolisms) [14, 34–36]. The high prevalence of prophages at vents are attributed to the geochemical dynamism of these habitats, which facilitates horizontal gene transfer (HGT), resulting in a broader functional gene set that may increase microbial adaptability to the extreme environmental fluctuations [37, 38]. Although prophages are abundant in free-living microbes at vents [35], not much is known about the abundance, diversity, or role of phages in influencing symbiotic microbes. Nonetheless, interest in the topic has grown rapidly, with recent studies from deep-sea chemosynthetic symbioses harboring mixed results regarding the presence of prophages and the possibility of an AMG-mediated phage-microbe-animal tripartite symbiosis [37, 39–42]. Recently, it has been suggested that deep-sea tubeworms may activate prophages embedded in their symbionts’ genomes as a mechanism to control symbiont populations and regulate nutrient release [43]. Furthermore, recent population genomic studies suggest bacteriophages may also influence strain-level population structuring of microbial symbionts from deep-sea hydrothermal vents [21, 22].

**Figure 1:**
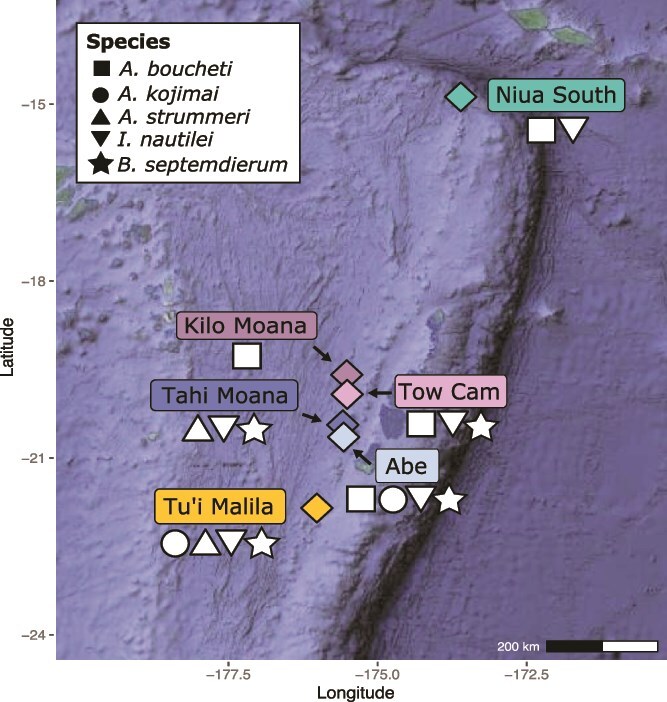
Map of the Lau Basin hydrothermal vent fields from which animal specimens were collected for this study. Vent field color scheme used in this figure is consistent through all downstream figures. Figure adapted from Breusing *et al.* 2022 [21].

This study investigates phages associated with the chemosynthetic bacterial symbionts of foundation mollusk species at six deep-sea hydrothermal vent fields separated by 10s to 100 s of kilometers in the Lau Basin (Tonga) (Fig. 1). Specifically, we investigated evidence for interaction with phages in the chemosynthetic symbionts of IUCN Red-List Endangered and Vulnerable (https://www.iucnredlist.org) sympatric snails *Alviniconcha boucheti* [44], *Alviniconcha kojimai* [45], *Alviniconcha strummeri* [46], and *Ifremeria nautilei* [47], as well as the mussel *Bathymodiolus septemdierum* [48]. Each snail or mussel species harbors 1–2 specific intracellular endosymbiont species in their gill tissue, with gammaproteobacterial symbionts *Candidatus* Thiodubiliella endoseptemdiera (Family *Thioglobaceae*) in *B. septemdierum,* distinct strains of gamma1 (Family *Thiomicrospiraceae*) in *A. kojimai* and *A. strummeri*, respectively, GammaLau (Family *Sedimenticolaceae*) in *A. strummeri*, thiotrophic SOX (Family *Arenicellaceae*) and methanotrophic MOX (Family *Methylomonadaceae*) in *I. nautilei*, and campylobacterial symbionts *Sulfurimonas Epsilon* and *Sulfurimonas* sp. in *A. boucheti* (Family *Sulfurimonadaceae*). Previous research on these symbionts have demonstrated that they exhibit strain-level differentiation by geography, with genomic evidence for locally adapted strains primarily driven by variation in vent fluid chemistry among vent fields as well as with potential viral interactions [2, 3], suggesting that symbionts interact with locally distinct viral assemblages according to geography [21, 22]. However, a recent analysis of environmental phage populations at hydrothermal vents in the Lau Basin showed that roughly half of the phage species are present across multiple vent locations [49]. Given this, we examined the lytic and lysogenic phage communities present in symbiont-containing tissues in symbiont genomes to generally survey the phages interacting with these symbionts to determine if there is evidence for variation in symbiont-phage interactions by symbiont species or geographic location.

## Materials and Methods

### Phage detection, species clustering, host prediction, and taxonomic classification

The 219 metagenomes (Table 1) and 200 high-quality symbiont MAGs used in this study (Table 2) were previously sequenced and assembled in Breusing *et al.*, 2022 and 2023 (Supplementary Table 1) [21, 50]. Prophages were identified in symbiont MAGs and metagenomes using VIBRANT v1.2.1 [51]. Phage identifiers were standardized to encode their source and key attributes. For phage sequences detected in metagenomes, IDs follow the structure: **[source metagenome]_[life cycle]_[scaffold].** For prophages identified directly within symbiont MAGs, the format is identical but includes an additional **“MAG”** tag to denote their genomic origin.

**Table 1:** Total number of metagenomes analyzed, and the average number of symbiont-targeting phage with ≥10kbp and ≥ 4 ORFs per Mbp of metagenome.

Host animal metagenome	No. metagenomes	Avg no. symbiont-targeting phages/Mbp	No. species clusters	No. symbiont-targeting species clusters
***I. nautilei***	59	0.00307	19	15
***A. boucheti***	23	0.00023	3	2
***A. kojimai***	17	0.01626	8	5
***A. strummeri***	22	0.00632	9	4
***B. septemdierum***	98	0.00475	70	59

**Table 2:** Total number of MAGs mined; total number, percent, and average number per Mbp of lysogenic phage (≥10kbp, ≥4 ORFs) found in each MAG as identified by VIBRANT; and total and average number per Mbp of CRISPR spacers found per symbiont type.

Symbiont MAG	No. MAGs	No. prophages	% MAGs with lysogenic phage	Avg no. prophage/Mbp	Total no. spacers	Average no. Spacers/Mbp
**SOX *- I. nautilei***	58	0	0	0	1157	4.5653
**MOX *- I. nautilei***	3	0	0	0	0	0.0000
**Epsilon *- A. boucheti***	8	0	0	0	32	2.3158
***Sulfurimonas - A. boucheti***	6	0	0	0	47	1.5991
**Gamma 1 *- A. strummeri***	19	4	21%	0.036	21	0.2957
**Gamma1 *- A. kojimai***	16	14	88%	0.203	215	3.4597
**Ca. T endoseptemdiera *- B. septemdierum***	92	0	0	0	1920	7.6743

To create species-level phage clusters, viral genomes ≥10 kb and ≥ 4 open reading frames (ORFs) were clustered with dRep v3.4.5 [53] using 95% Average Nucleotide Identity (ANI) and 50% minimum alignment. For both lytic and lysogenic phages from the metagenomes and prophages from the MAGs. Host predictions were performed only for lytic viruses using iPHoP v1.3.3 with default parameters [52]; lytic phages may originate from either symbiont-associated or non-symbiotic taxa, whereas lysogenic phages are unambiguously defined by their integration within symbiont genomes and were, therefore, excluded from the iPHoP analysis [53]. We used the custom database function of iPHoP to create a database of symbiont MAGs for host prediction. Lytic phages identified with ≥90% confidence were considered symbiont-targeting phage, since this threshold yields high-quality host-phage matches [54]. If at least one phage within a cluster was identified as symbiont-targeting, we inferred that this trait applied to the entire cluster, reflecting a likely shared biological capacity including host range, as has been observed previously [54].

To visualize the phage clusters, we used the cluster dendrogram generated with dRep. We circularized the dendrogram by modifying the radialtree code and integrated it into the dRep code (Original radialtree: https://github.com/koonimaru/radialtree/blob/main/radialtree.py, Original dRep: https://github.com/MrOlm/drep/blob/master/drep/d_analyze.py, forked files in https://github.com/michellehauer/Lau_Basin_09-16_Phage_Analysis/blob/main/drep_fork/) using Python v3.8.9. The relative frequency of each symbiont-targeting phage species cluster was calculated as the proportion of phages in each cluster found at a given location relative to the total number of phages in that cluster and was represented as a bubble plot using ggplot2 [55] in RStudio v2023.12.1 + 402. Phage taxonomic classification was performed using geNomad v1.6.1 [56] with default parameters, and only classifications with ≥5 gene alignment and an agreement of ≥80% were retained for downstream analysis.

### Investigation of genetic elements contributing to symbiotic functionality in symbiont MAGs

We screened prophages for AMGs using VIBRANT [51] and only retained AMGs flanked by viral genes, viral-like genes, or hypothetical genes of likely viral origin (based on V-scores). For AMGs detected in a prophage that belonged to a dRep species cluster, a blastp v2.101 analysis was performed to query the AMG protein sequence against a database of all prophage protein sequences from that prophage species cluster, followed by a MAFFT v7.505 alignment.

### CRISPR spacers in symbiont MAGs

CRISPR spacer abundance and diversity were determined by MinCED v0.4.2 using default parameters (https://github.com/ctSkennerton/minced). Spacer taxonomic classifications were determined by a blastn analysis of CRISPR spacer sequences against the nt virus database using blast-plus v2.13.0 and Python 3.11.6 [57] . We defined a CRISPR spacer “match” as having 100% identity across ≥20 bp, parameters shown to be suitable for determining the species or genus from which the spacer derived [16]. Plasmid sequences were removed by screening spacers against the PLSDB (Plasmid Database, v2023_11_03_v2) [58] using MASH Screen and default parameters of max p-value 0.1 and percent identity 99% on the web user interface. To determine whether symbionts harbor spacers that match phages found in metagenomes, a reciprocal blast analysis with parameters -word_size 20 and -max_target_seqs 1 [57] was performed using the “best hit” phage per species cluster as determined by dRep. Results with ≥97% percent identity and ≤1 mismatch were considered a match.

## Results

### Symbiont-containing animal gill tissues harbor distinct bacteriophage viromes that do not exhibit strict geographic endemism

**Figure 2:**
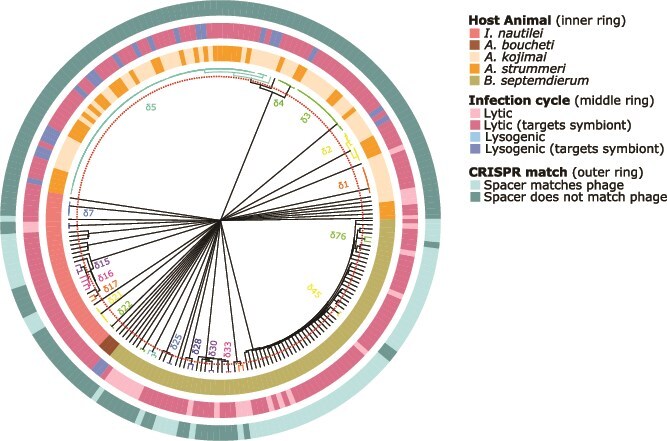
Circle dendrogram representing all lytic and lysogenic phage collected across all metagenomes. Each branch corresponds to a phage, with species clusters defined by 95% ANI, indicated by the red dotted line. These species clusters are color-coded for enhanced clarity, and black branches denote singletons. dRep does not calculate or visually represent branch relationships for sequences with less than 88% ANI. The innermost ring illustrates the source animal metagenome from which the phage was derived. The middle ring represents the phage’s cycle and indicates whether it was predicted to target the symbiont; if at least one individual within a cluster was identified as symbiont-targeting, the entire cluster was designated as such. The outermost ring is a binary ring that signifies that a CRISPR spacer from one of the symbiont MAGs matched to that phage sequence.

A total of 5161 individual phages, averaging 0.0059 phage per megabase pair (Mbp), were identified across the 219 metagenomic samples, but only 222 met our quality threshold metrics requiring ≥10 kb length and ≥4 open reading frames (Fig. 2, Supplementary Table 2); a summary of the number of MAGs and metagenomes analyzed per species, along with a summary of results, is provided in Supplementary Figs 1A–E. Consistent with prior large-scale viromic surveys showing that most viral genomes assemble only partially due to extensive genomic diversity and mosaicism [59], the majority of viral contigs recovered here are low-quality, which is typical for metagenome-derived viral datasets (Supplementary Table 3). These 222 phages represented a total of 105 species-level phage clusters, though 84 (80%) of these species comprised of a single, unique phage sequence (i.e. a “singleton”) detected only in one snail or mussel’s metagenome and did not form a species-level cluster with any other sequence (Fig. 2, Supplementary Tables 4 and 5). Of these 222 phages, 180 (81.08%) were predicted to target the symbionts (Fig. 2, Supplementary Tables 4–6) and, subsequently, 91 species clusters were designated as symbiont-targeting (Fig. 2, Supplementary Table 6).

Except for four phage species clusters present in both the *A. kojimai* and *A. strummeri* metagenomes, most phage species clusters were uniquely observed in the metagenome of only one host animal species, suggesting that distinct bacteriophage viromes infect their respective microbiomes (Fig. 2, Supplementary Table 4). Four phage species clusters (φ2- φ5) were shared between *A. kojimai* and *A. strummeri*, though only φ3, φ4 and φ5 targeted the gamma1 symbiont (Supplementary Tables 4, 6). Phage species clusters φ4 and φ5 were both predicted to contain a mix of lysogenic and lytic forms (Fig. 2, Supplementary Table 2), whereas φ3 was exclusively lytic.

**Figure 3:**
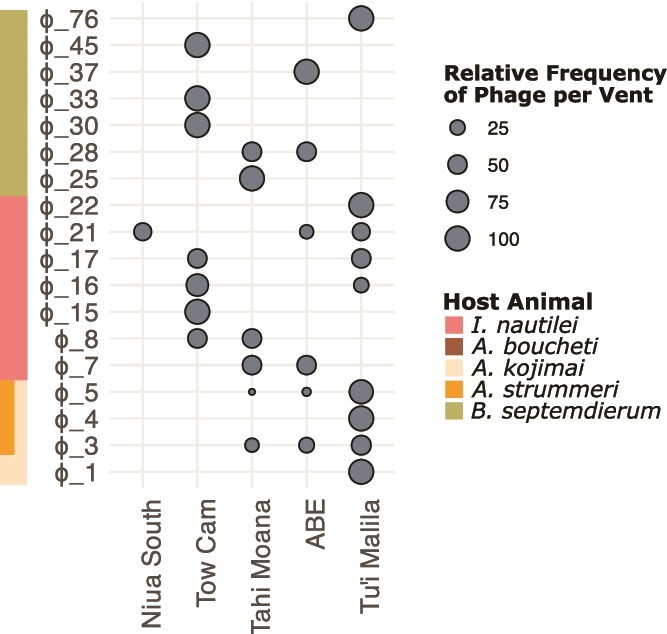
Bubble heatmap representing the proportional distribution of the occurrence of each phage species cluster in the metagenomes by hydrothermal vent field (ordered north to south). Only the phage species that were detected in more than one individual metagenome were included (i.e. excludes singletons).

Across all host animal metagenomes, phage species did not consistently exhibit strict geographic endemism at vents within the Lau Basin (Fig. 3, Supplementary Table 4). Of the 18 total symbiont-targeting phage species clusters that were identified in more than one individual metagenome, 10 were restricted to a single vent field (e.g. φ1 in *A. kojimai,* φ4 in *A. kojimai* and *A. strummeri* metagenomes, φ15 and φ22in *I. nautilei*, and φ25, φ30, φ33, φ37, φ45, φ76 in *B. septemdierum*). The other 8 were associated with the same host species across two or more Lau Basin vent fields.

### Prophages found exclusively in gamma1 symbionts

Prophages meeting our minimum length threshold of 10 kb were only detected in the gamma1 symbiont MAGs of *A. kojimai* and *A. strummeri* (Fig. 2, Supplementary Fig. 2, Supplementary Tables 1, 7, 8), though two shorter prophages were present in just two of the Ca. T. endoseptemdiera genomes, but were not considered for further analyses. Among the gamma1 symbionts from *A. kojimai* and *A. strummeri,* 12/16 (75%) and 4/19 (21%) harbored one or more prophage, representing an average of 0.19 and 0.049 prophage/Mbp, respectively (Supplementary Table 1).

Of the gamma1-*A. kojimai* prophages, 10 were found in individuals from Tu’i Malila and 2 at ABE; all 4 gamma1-*A. strummeri* MAGs were found at Tu’i Malila (Fig. 3, Supplementary Fig. 2). These prophages formed two distinct species clusters (95% ANI)— φ4 and φ5—but three distinct strains (99% ANI) (Supplementary Fig. 2, Supplementary Tables 8 and 9). Species cluster φ4 was found in a single *A. strummeri*–gamma1 MAG from Tu’i Malila, and the remaining 15 prophages belong to species cluster φ5. Within φ5, gamma1-*A. kojimai* gamma1 symbionts from ABE formed a distinct strain-level group, while both *A. strummeri* and *A. kojimai* gamma1 prophages from Tu’i Malila formed another distinct strain. These results may indicate that prophage species, like the phages in the metagenomes, do not exhibit strict species-level geographic endemism; however, they may segregate at the strain-level by vent field.

### Phages predominantly belong to the class *Caudoviricetes*

Of the taxonomically classifiable phages and CRISPR spacers, most species were from the dsDNA class *Caudoviricetes* (Supplementary Tables 4, 7, 10), a common and highly diverse marine phage family previously reported to be abundant at hydrothermal vents [36]. Three lytic *Caudoviricetes* phage clusters from Tow Cam—φ30, φ32, and φ34—were classified within the order *Schitoviridae*. Only one individual lytic phage—φ104 at Tu’i Malila—was of the class *Malgrandaviricetes* in the order *Microviridae*, a ssDNA bacteriophage also commonly found in hydrothermal vent ecosystems including the Lau Basin [34, 36]. It is interesting to note that the CRISPR spacers encompassed a greater diversity of viral origins than *Caudoviricetes*, spanning multiple additional classes such as *Arfiviricetes*, *Ellioviricetes*, *Malgrandaviricetes*, *Megaviricetes*, and *Revtraviricetes*. This suggests a greater diversity of historic infections than *Caudoviricetes*, or a high level of genomic exchange between bacterial symbionts and viruses.

### Limited AMG occurrence in symbiont-infecting prophages

Prophage species φ5 infecting gamma1 from *A. kojimai* (K09_101 at ABE) was predicted to encode an AMG. However, this AMG was annotated as glutamine-fructose-6-phosphate transaminase (*glmS*) (Supplementary Table 11), a gene i.e. involved in UDP-GlcNAc synthesis, a precursor of peptidoglycan for bacterial cell wall formation, which is not clearly auxiliary. Furthermore, the blastp analysis of the *glmS* gene from K09_101 did not have any reliable matches or alignments to the other protein sequences from individual phages in species cluster φ5, providing no substantial evidence for widespread occurrence of this *glmS* gene among the chemosynthetic symbionts examined here. No other prophages contained genes meeting our criteria for bona fide AMGs.

### CRISPR spacers potentially indicate immune system mechanisms in SOX and Ca. T. endoseptemdiera

Among all MAGs, 58% contained at least one CRISPR spacer, with counts ranging from 1 to 230 per MAG (Supplementary Table 12), and no spacers were identified as plasmid derived. No spacers were identified in I. nautilei MOX symbionts, whereas Ca. T. endoseptemdiera (B. septemdierum) symbionts harbored the greatest average number of CRISPR spacers per Mbp and gamma1 symbionts had the lowest average number of spacers per Mbp (Table 2, Supplementary Table 12).

Some CRISPR spacers found in the SOX symbionts of *I. nautilei* and the Ca. T. endoseptemdiera symbionts of *B. septemdierum* matched symbiont-targeting lytic phages that were present in the metagenome and, therefore, have the potential to represent active immune systems (Fig. 2, Supplementary Table 13). Specifically, 66 lytic phage-spacer matches were found in SOX and 280 lytic phage-spacer matches were found in the Ca. T. endoseptemdiera symbiont MAGs. In some cases, many CRISPR spacers (up to 27) matched to a single phage sequence.

## Discussion

Here, we investigated the lytic and lysogenic phages associated with symbiont-containing gill tissues of five co-occurring, endangered, and vulnerable deep-sea hydrothermal snail or mussel species from hydrothermal vents in the Lau Basin. In summary, our results indicated that lytic phages present in the gill tissues primarily targeted the dominant bacterial symbionts in that tissue, and that prophages and associated AMGs were not detected in most symbiont genomes. The high proportion of symbiont-targeting phage species suggests that the symbionts may interact with a large diversity of phages. The smaller number of phage species that were consistently detected across multiple metagenomes or symbiont genomes exhibited host-specificity at the species- (but not strain) level and did not exhibit strict geographic endemism among hydrothermal vent fields in the Lau Basin.

Whereas some phages are generalists and cosmopolitan, most have high host-specificity at the species-level [60] or even at the strain-level [61–64], and vent-dwelling phages have typically been found to be predominantly endemic at large geographic scales [34, 65]. Previous population genomics work on the chemosynthetic symbionts of mollusks from the Lau Basin showed that they exhibit geographic population structure that, based on variation in gene content, could be partially driven by interactions with locally distinct phage communities [21, 22]; we, therefore, expected to observe unique vent-endemic phage infections among our symbiont strains based on geographic location. However, the abundance of singletons makes it difficult to determine whether the symbionts are consistently interacting with location-specific (endemic) phage communities. It is notable that some symbiont-targeting phage species are shared between the two southernmost vent fields, ABE and Tu’i Malila, which are ~141 km apart, or even between the northernmost vent field, Niua South, and the southernmost vent field, Tu’i Malila, which are ~821 km apart. Although phage infections of SOX and gamma1 were not predominantly site-specific, five of the six phage species clusters infecting Ca. T endoseptemdiera symbionts of *B. septemdierum* (φ25, φ30, φ33, φ45, φ76), did indeed appear to be location-specific. One exception, φ28, was found at both Tahi Moana and ABE, which is likely a consequence of the proximity of the two vent fields. These results suggest that populations of Ca. T endoseptemdiera may interact with location-specific phage communities.

Given that these symbiotic mollusks typically harbor one or two dominant bacterial symbiont species at high cell densities in their gill tissues, it is unsurprising that most of the lytic phages in the tissue samples were predicted to infect the symbionts. These symbionts predominate the gill tissue microbiomes, and although their abundance and density have not been directly assessed, a similar vent mussel species, *Bathymodiolus puteoserpentis,* has been estimated to harbor a mean abundance of 2.5 × 10^12^ chemosynthetic bacterial symbionts per specimen [66]. Whereas most phage species were found exclusively in one host animal metagenome, *A. strummeri* and *A. kojimai* had more similar bacteriophages, and these were largely predicted to infect gamma1 symbionts, the bacterial symbiont that these snail species share. This finding suggests that animals typically harbor distinct phage viromes, underscoring the specificity of these phages [60–64].

The broad taxonomic diversity of CRISPR spacers indicates that symbiont lineages may have historically encountered a range of viral groups beyond the *Caudoviricetes* that were predominantly detected in the phage communities. Since CRISPR arrays retain a long-term record of infection events, they can capture exposure to transient, rare, or low-abundance phages that may not be present or detectable in current viral communities. Many of the CRISPR spacers matched lytic phage sequences in *I. nautilei* and *B. septemdierum* metagenomes, including those that were predicted to infect their respective symbionts, suggesting that not all the CRISPR spacers are necessarily representative of historic infections but may also reflect contemporary interactions. In contrast, the gamma1 spacers did not match any active infections, and therefore gamma1 spacers likely only represent historic infections. Additionally, the relatively low number of CRISPR spacers found in gamma1 compared to the other symbiont species may suggest that their CRISPR-Cas immune system may be less active. Indeed, the gamma1 symbionts were the only species in this study to harbor detectable prophages but also had the lowest average number of CRISPR spacers. Whether this lack of spacer protection is an evolutionary advantage, since it may more readily allow for the integration of beneficial prophages, is unknown. If prophage infections are indeed advantageous for gamma1 symbionts—whether through AMGs, HGT [67, 68], or super immunity [69, 70]**—**or if they may benefit the host animal through population control or nutrient release upon lytic activation [71, 72], as has been hypothesized for a deep-sea tubeworm [42], then maintaining a CRISPR immune system that counteracts prophage activity may impose a fitness cost. Instead, gamma1 symbionts may employ other mechanisms to prevent infection from unwanted invaders, e.g. via restriction modification genes or directly through the super immunity potentially provided by prophage integration. This may also provide insight into the growth rates of the bacterial symbionts, in that faster growing microbes have been observed to harbor diminished CRISPR-Cas immunity and greater number of prophage relative to slower growing bacteria [73].

The lack of detected prophages in all other symbiont species was unexpected, considering their prevalence in vent bacteria [32, 33], *Gammaproteobacteria* generally [43], and previous findings of prophages in hydrothermal vent symbionts including mussels, sponges, and clams [42]. It is notable that prophages were detected in two Ca. T. endoseptemdiera genomes, but both were shorter than our 10 kb inclusion threshold and were, therefore, excluded from further analyses. Although the limited identification of prophages could be an artifact of methodology, other studies of chemosynthetic symbionts also found limited evidence for prophages [40].

The absence of detected prophages in most symbiont genomes might be explained by the fact that intracellular symbionts are sheltered from the extreme chemical and temperature dynamism of hydrothermal vents, whereas free-living environmental microbes may confer beneficial fitness consequences from prophages, as the resulting genetic augmentation could confer adaptive advantages critical for survival in such fluctuating environments [12, 24–31]. From a game theoretical perspective, the strategy of free-living vent microbes accepting prophages can be seen as a form of genetic hedging [74–76], wherein the potential long-term benefits of acquiring new genes outweigh the costs (e.g. lysogenic induction into the lytic cycle). Conversely, microbes living within host cells face different strategic considerations wherein the relative environmental stability reduces the benefit of such genetic diversity, and the sudden activation of a lytic cycle could lead to rapid and unchecked microbial cell death, disrupting the symbiotic relationship.

Our data provided no clear evidence of prophages being fundamentally involved in the bacterial-animal symbioses at deep-sea hydrothermal vents of the Lau Basin via AMGs. This may be an artifact of the methodology, e.g. detection limits, genome fragmentation, or the absence of intact lysogenic prophages in symbionts, although prophages with AMGs are more common in habitats where metabolic modification is critical for survival, and, therefore, habitats that are nutrient enriched typically do not harbor prophage with AMGs [69]. The overall stability of the host-associated conditions, coupled with likely high nutritional availability, may explain the limited detection of AMGs. Furthermore, not all beneficial prophages are involved in modifying their host’s metabolic pathways; some may primarily provide benefits like increased resistance to superinfection, e.g. through lysogenic allelopathy [69, 70], or HGT [67, 68]. The temperate phage species clusters in gamma1 symbionts could also act as population control, protecting the host animal from the destabilizing effects of symbiont over-colonization [77].

## Conclusions

In this study, we investigated the phage content in metagenomes from the symbiont-containing gill tissues of four hydrothermal snail and one mussel species, as well as the prophage and CRISPR spacer content in their bacterial symbiont MAGs, across a ~821 km range in the Lau Basin. The complex interplay between phages and chemosynthetic symbionts in deep-sea hydrothermal vent ecosystems underscores a multifaceted ecological dynamic that may significantly impact microbial population dynamics. Our study revealed that contemporary interaction with lytic and lysogenic phages do not show a geographic pattern, though we found some evidence for historical infections by strain-specific phages that vary by location. To better understand whether genetic contributions from phages through HGT influence symbiont adaptability and strain-level variation, further research examining symbiont genomes for genes of phage origin (e.g. virulence genes) and expression is warranted. Furthermore, temporal analyses may elucidate the stability of these phage infections and dynamics of symbiont-phage coevolution, providing more clues as to whether some of these phage infections confer fitness consequences. Microscopy of host animal tissue may also provide clarity regarding whether the observed lytic phages in this study are found in the intracellular symbiont populations within the gill tissue or outside on the surface of the gills, which may further influence the interpretation of these interactions.

Given that all five animals in this study are currently classified as “Endangered” or “Vulnerable” on the IUCN Red List (https://www.iucnredlist.org) and play a vital role in the ecology of hydrothermal vents, understanding the factors influencing the success and connectivity of their symbionts is crucial for future conservation and management efforts. Our results indicate that phages may play a multifaceted role in controlling symbiont populations and bacterial-animal symbiotic dynamics, including potential bacterial host death through lytic infection. These interactions may thereby impact the ecological stability of these hydrothermal vent communities by preventing overdominance of particular symbiont strains or modulating the fitness of the locally available symbiont strains. Elucidating the role of phages in host-symbiont dynamics may, therefore, be an integral component of informing effective future management strategies.

## Supplementary Material

Supplementary_Figures_ycag022

Supplementary_Tables_Revised_2_ycag022

## Data Availability

The previously published raw metagenomic reads as well as the corresponding MAG assemblies are available at the National Center for Biotechnology Information under BioProject PRJNA855930 and PRJNA523619. All bioinformatic scripts are published to https://github.com/michellehauer/Lau_Basin_09-16_Phage_Analysis
