# Supplementary material for: Phage-microbe interactions may contribute to the population structure and dynamics of hydrothermal vent symbionts
Source: ISME Commun. 2026 Feb 3;6(1):ycag022. doi: 10.1093/ismeco/ycag022 (PMC12927875; doi:10.1093/ismeco/ycag022)
Supplement: Supplementary_Figures_ycag022 [file supplementary_figures_ycag022.pdf]

Supplementary Figures

Phage-microbe interactions may contribute to the population structure and dynamics of hydrothermal vent symbionts

Michelle A. Hauer[1,2,3], Katherine M. Klier[4,5], Marguerite V. Langwig[4,5], Karthik Anantharaman[4,6,7], Roxanne A. Beinart[1*]

[1]Graduate School of Oceanography, University of Rhode Island, Narragansett, RI, USA
[2]School of Marine and Environmental Affairs, University of Washington, Seattle, WA, USA
[3]Northwest Fisheries Science Center, National Oceanic and Atmospheric Administration, Seattle, WA, USA
[4]Department of Bacteriology, University of Wisconsin-Madison, Madison, WI, USA
[5]Freshwater and Marine Sciences Program, University of Wisconsin-Madison, Madison, WI, USA
[6]Department of Integrative Biology, University of Wisconsin-Madison, Madison, WI, USA
[7]Department of Data Science and AI, Wadhwani School of Data Science and AI, Indian Institute of Technology Madras, Chennai, Tamil Nadu, India
***Corresponding author**: Roxanne A. Beinart, rbeinart@uri.edu

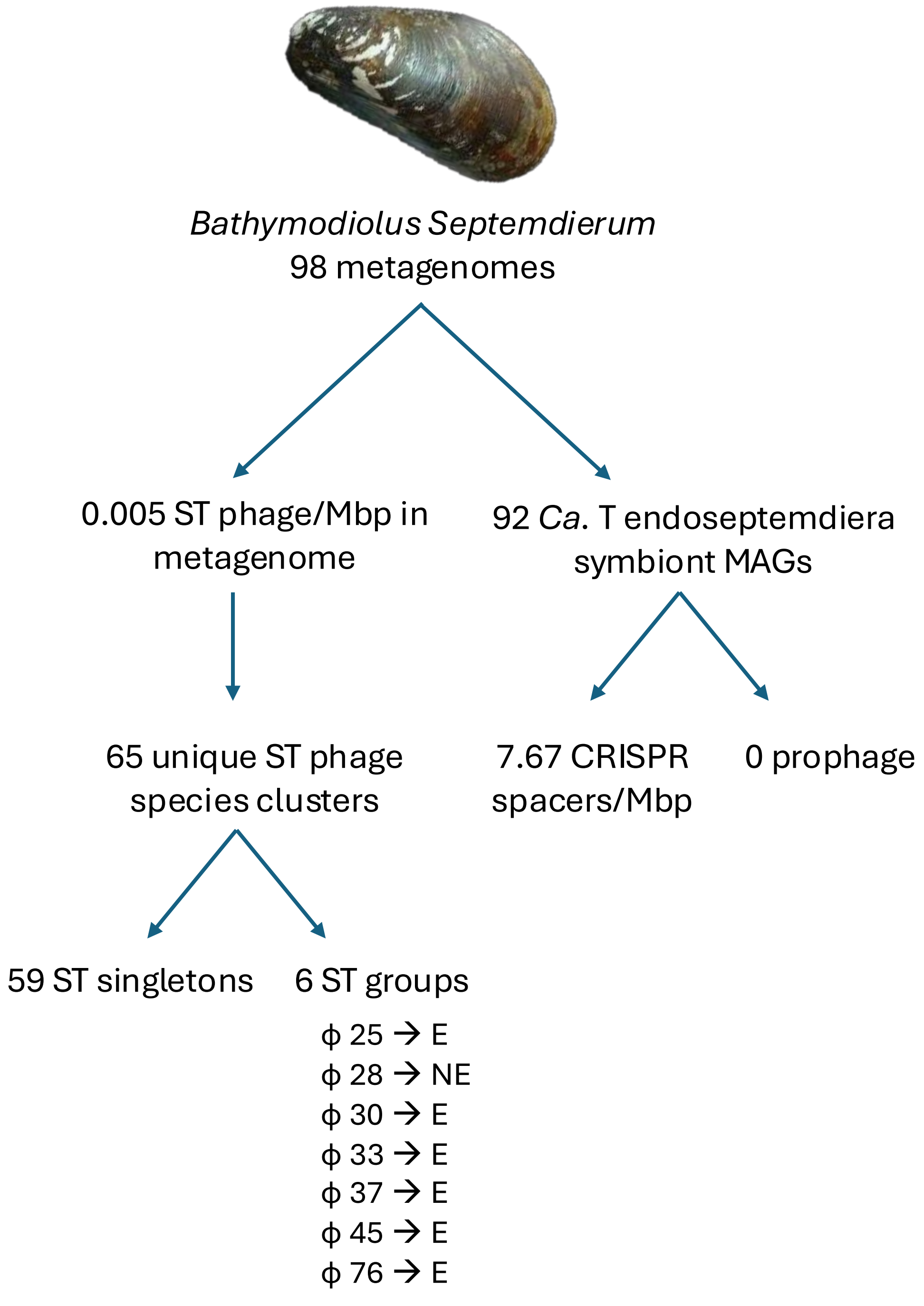

Bathymodiolus Septemdierum
98 metagenomes
0.005 ST phage/Mbp in metagenome
92 *Ca.* T endoseptemdiera symbiont MAGs
65 unique ST phage species clusters
7.67 CRISPR spacers/Mbp
0 prophage
59 ST singletons
6 ST groups
ϕ 25 → E
ϕ 28 → NE
ϕ 30 → E
ϕ 33 → E
ϕ 37 → E
ϕ 45 → E
ϕ 76 → E

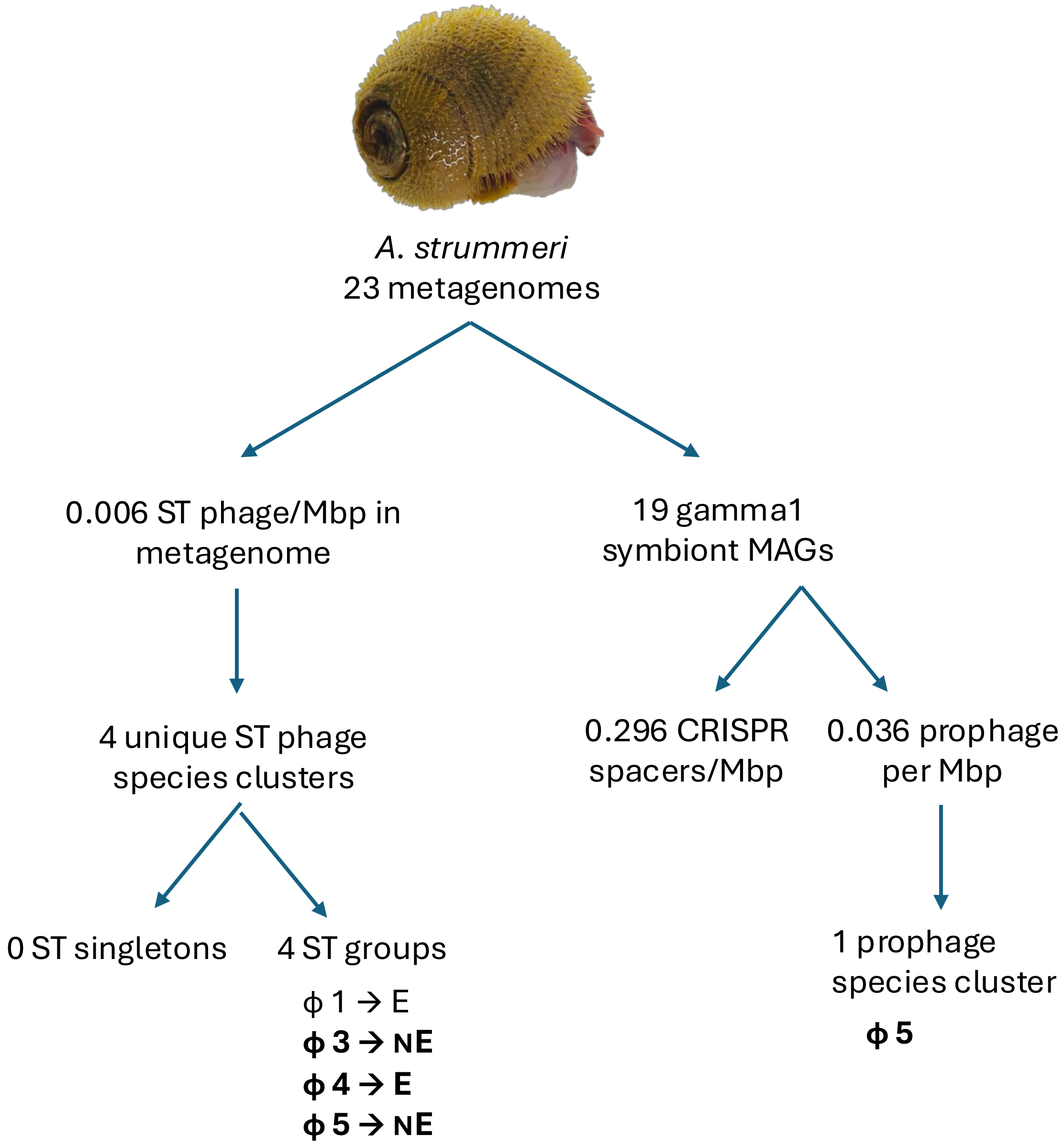
A. strummeri
23 metagenomes
0.006 ST phage/Mbp in metagenome
19 gamma1 symbiont MAGs
4 unique ST phage species clusters
0.296 CRISPR spacers/Mbp
0.036 prophage per Mbp
0 ST singletons
4 ST groups
ϕ 1 → E
ϕ 3 → NE
ϕ 4 → E
ϕ 5 → NE
1 prophage species cluster
ϕ 5

1b.

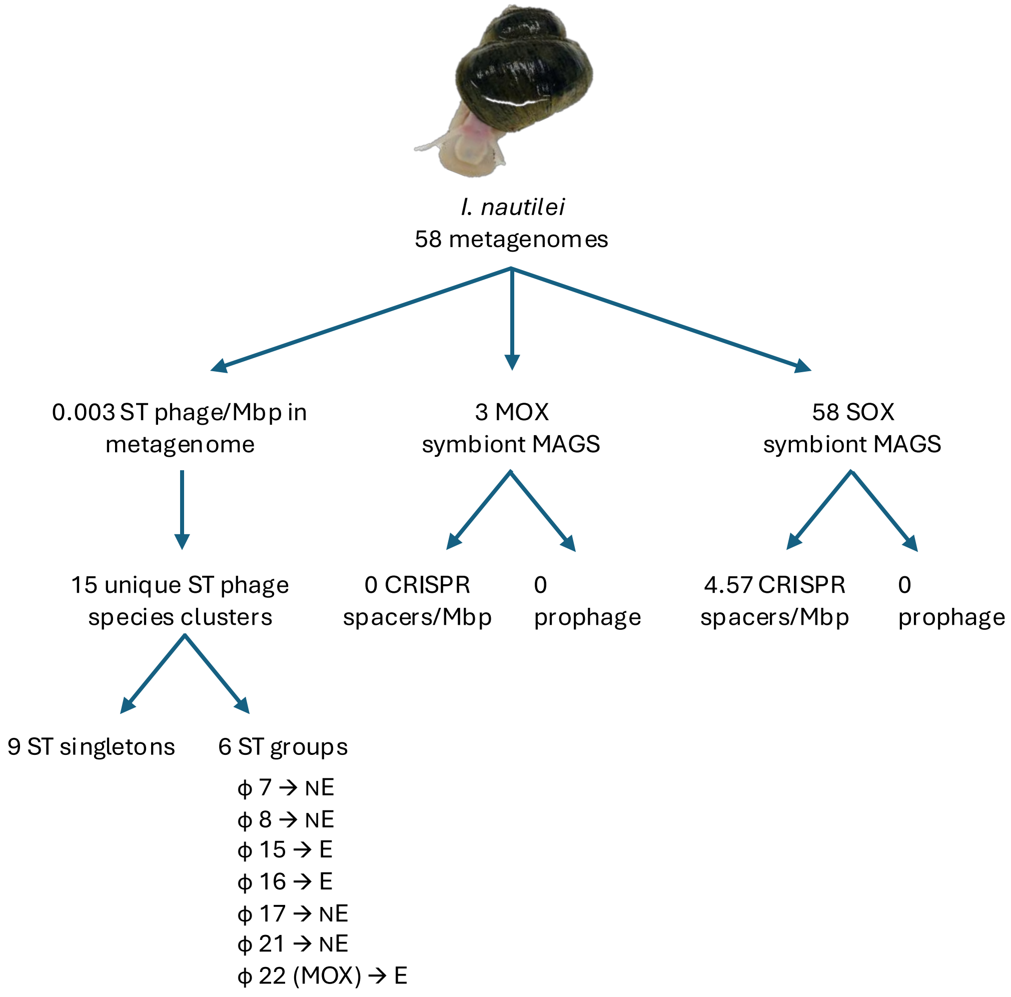

I. nautilei
58 metagenomes
0.003 ST phage/Mbp in metagenome
3 MOX symbiont MAGS
58 SOX symbiont MAGS
15 unique ST phage species clusters
0 CRISPR spacers/Mbp
0 prophage
4.57 CRISPR spacers/Mbp
0 prophage
9 ST singletons
6 ST groups
ϕ 7 → NE
ϕ 8 → NE
ϕ 15 → E
ϕ 16 → E
ϕ 17 → NE
ϕ 21 → NE
ϕ 22 (MOX) → E

1c.

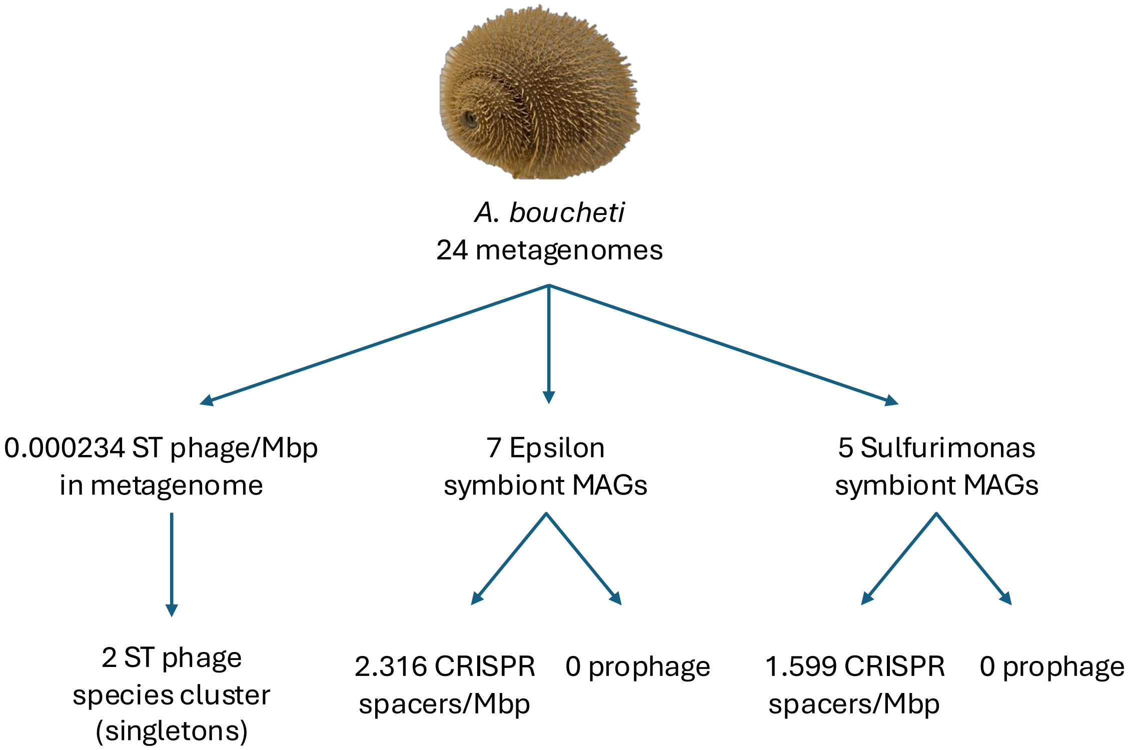

A. boucheti
24 metagenomes
0.000234 ST phage/Mbp
in metagenome
7 Epsilon
symbiont MAGs
5 Sulfurimonas
symbiont MAGs
2 ST phage
species cluster
(singletons)
2.316 CRISPR
spacers/Mbp
0 prophage
1.599 CRISPR
spacers/Mbp
0 prophage

1d.

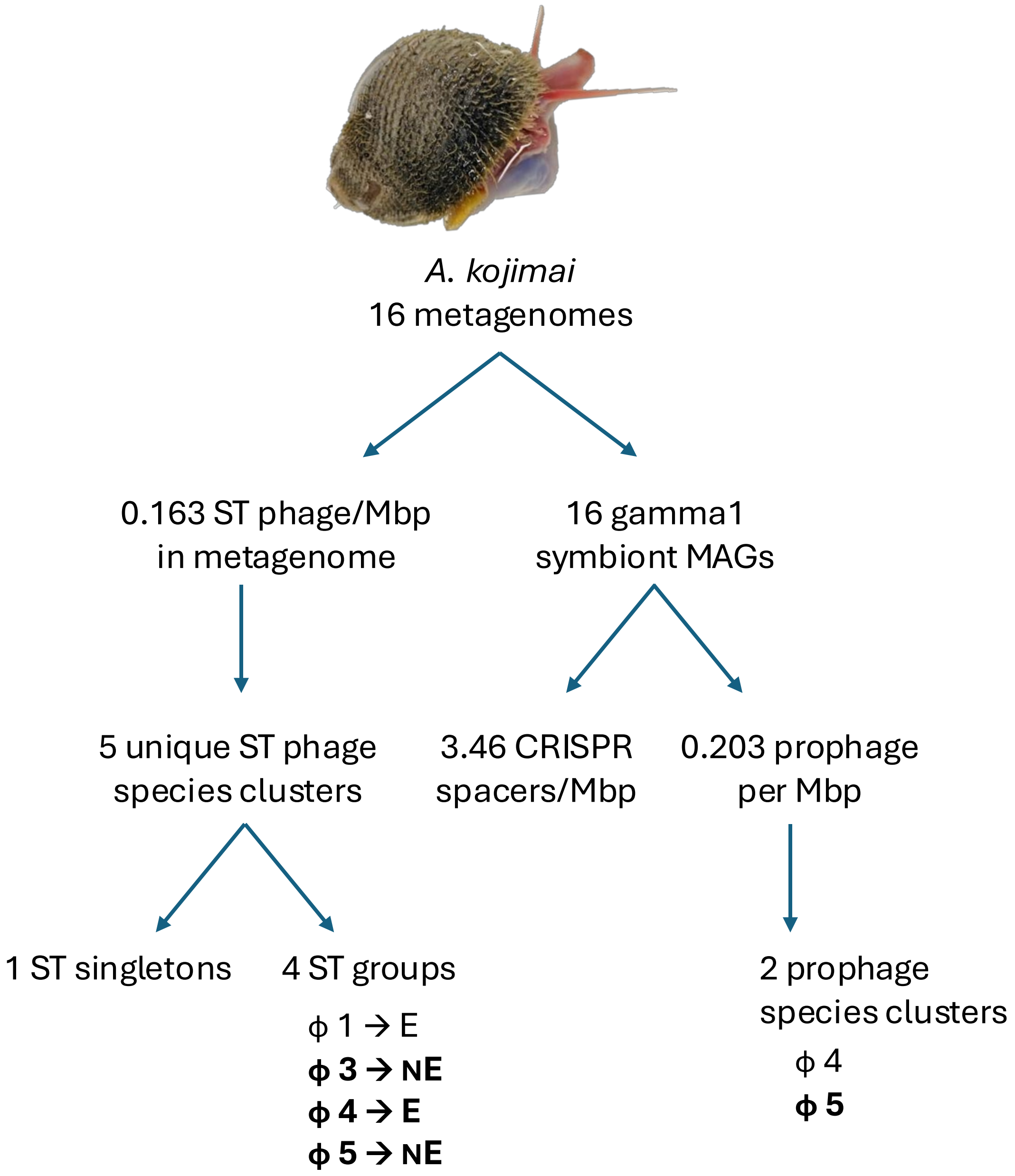

1e.

Supplementary Figures 1a-1e. Summary of phage detections across symbiont MAGs, metagenomes, and CRISPR spacers for each symbiont investigated in this study. Values indicate prophage abundance (per Mbp), lytic phage abundance (per Mbp), and CRISPR spacer density (per Mbp), along with the number of unique species clusters and singletons recovered. Bolded phage species clusters within *A. kojimai* and *A. strummeri* summaries indicate those which were found in tissues from both species. "E" and "NE" indicate endemic and not-endemic, respectively.

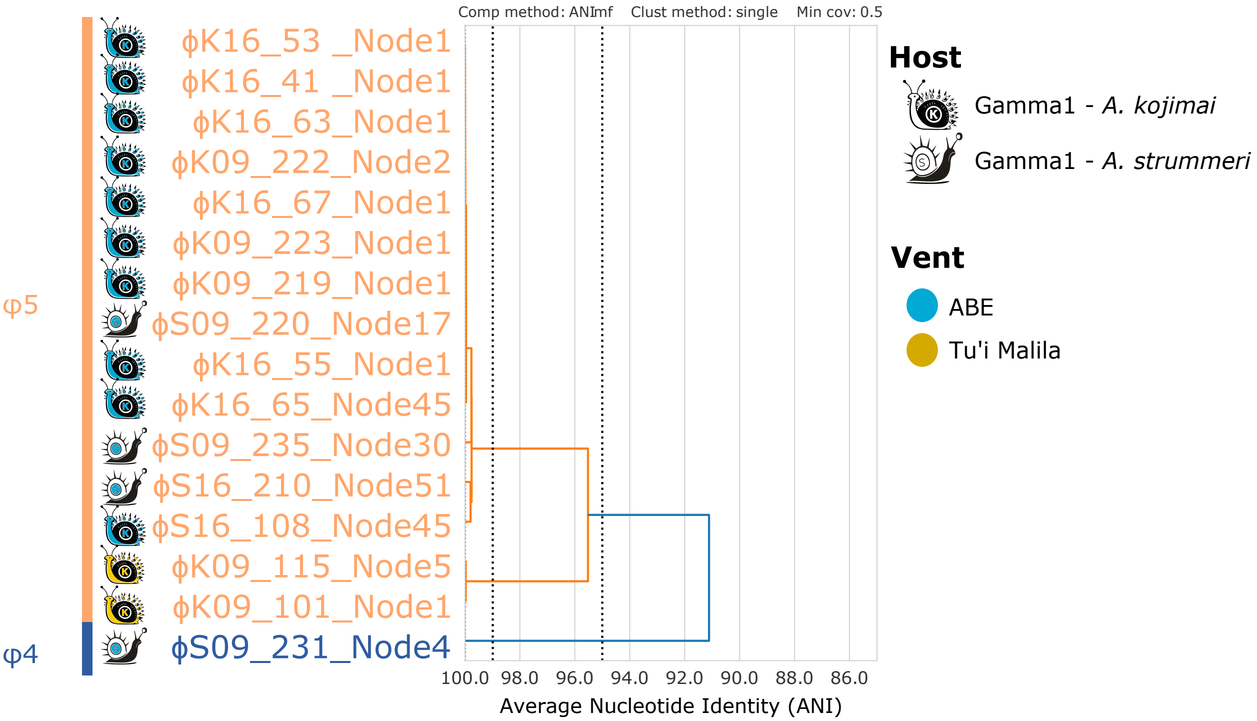

Supplementary Figure 2. Dendrogram representing all the prophages identified in the MAGs. *A. kojimai* and *A. strummeri* icons in blue represent those collected from ABE while those in yellow were collected from Tu'i Malila. Phages belonging to cluster φ5 are delineated in orange while φ4 are in dark blue. Species-level and strain-level clusters are delineated with a dotted line at 95% and 99% ANI, respectively.
